# Collagen-Heparin-FGF2-VEGF Scaffolds Induce a Regenerative Gene Expression Profile in a Fetal Sheep Wound Model

**DOI:** 10.1007/s13770-024-00667-9

**Published:** 2024-08-31

**Authors:** Merel Gansevoort, Corien Oostendorp, Linde F. Bouwman, Dorien M. Tiemessen, Paul J. Geutjes, Wout F. J. Feitz, Toin H. van Kuppevelt, Willeke F. Daamen

**Affiliations:** 1 Department of Medical BioSciences, Research Institute for Medical Innovation, Radboud University Medical Center, PO Box 9101, 6500 HB Nijmegen, The Netherlands; 2 Department of Urology, Research Institute for Medical Innovation, Radboud University Medical Center, PO Box 9101, 6500 HB Nijmegen, The Netherlands; 3 Present Address: HAN University of Applied Sciences, Arnhem, The Netherlands; 4 Present Address: Leiden University Medical Center, Leiden, The Netherlands; 5 Present Address: Canisius Wilhelmina Hospital, Nijmegen, The Netherlands

**Keywords:** Tissue engineering, Skin regeneration, Fetal wound healing, Collagen scaffold, Extracellular matrix

## Abstract

**Background:**

The developmental abnormality spina bifida is hallmarked by missing tissues (e.g. skin) and exposure of the spinal cord to the amniotic fluid, which can negatively impact neurological development. Surgical closure of the skin *in utero* limits neurological damage, but in large defects this results in scarring and contractures. Stimulating skin regeneration *in utero* would greatly benefit treatment outcome. Previously, we demonstrated that a porous type I collagen (COL) scaffold, functionalized with heparin (HEP), fibroblast growth factor 2 (FGF2) and vascular endothelial growth factor (VEGF) (COL-HEP/GF) improved pre- and postnatal skin regeneration in a fetal sheep full thickness wound model. In this study we uncover the early events associated with enhanced skin regeneration.

**Methods:**

We investigated the gene expression profiles of healing fetal skin wounds two weeks after implantation of the COL(-HEP/GF) scaffolds. Using laser dissection and microarrays, differentially expressed genes (DEG) were identified in the epidermis and dermis between untreated wounds, COL-treated wounds and wounds treated with COL-HEP/GF. Biological processes were identified using gene enrichment analysis and DEG were clustered using protein–protein-interaction networks.

**Results:**

COL-HEP/GF influences various interesting biological processes involved in wound healing. Although the changes were modest, using protein–protein-interaction networks we identified a variety of clustered genes that indicate COL-HEP/GF induces a tight but subtle control over cell signaling and extracellular matrix organization.

**Conclusion:**

These data offer a novel perspective on the key processes involved in (fetal) wound healing, where a targeted and early interference during wound healing can result in long-term enhanced effects on skin regeneration.

**Supplementary Information:**

The online version contains supplementary material available at 10.1007/s13770-024-00667-9.

## Introduction

The possibility to initiate prenatal closure of skin defects would greatly benefit care for the severe pathological condition spina bifida, where the back of the fetus fails to close over the spinal cord [1–3]. Prolonged exposure of the spinal cord to the amniotic fluid is detrimental to neurological development [4, 5], hence inducing fast closure of such defects by stimulating skin regeneration *in utero* may improve clinical outcomes. Biomaterials such as collagen-based scaffolds are excellent candidates to stimulate closure of defects by inducing skin regeneration [6]. In previous studies we found that the application of collagen (COL) scaffolds functionalized with heparin (HEP), fibroblast growth factor 2 (FGF2) and vascular endothelial growth factor (VEGF) promoted skin regeneration in sheep models of fetal wound healing. This model is particularly suited to investigate skin wound healing, even without various features associated with spina bifida—such as a lack of muscle tissue at the defect or chromosomal abnormalities. Nevertheless, this animal model provides a powerful platform to investigate skin regeneration *in utero*.

Generally, full thickness skin wounds of > 4 mm diameter in fetal sheep heal with a scar from a gestational age of ~ 80 days (term 140–147 days) [7]. The resulting scar tissue is contracted, with collagen organized in thick parallel bundles and a lack of skin appendages (e.g. hair follicles, sebaceous glands). When implanted into surgically made full-thickness skin wounds at 79 days gestation, COL-HEP-FGF2-VEGF (COL-HEP/GF) scaffolds lead to reduced wound contraction and enhanced the angiogenic response prenatally [8]. Postnatally, these scaffolds demonstrated superior skin regeneration at 1 and 6 months, as illustrated by an increase in the surface area of regenerated skin and evidence of hair follicle neogenesis [9].

The components used in these COL-HEP/GF scaffolds were carefully selected based on existing knowledge of their effects. The exogenous application of FGF2 has shown a positive effect on wound healing in several studies [10, 11] and classically FGF2 is known as an inhibitor of skin fibrosis through its prevention of myofibroblast activity [12, 13]. The growth factor VEGF stimulates angiogenesis during the proliferation phase and the ingrowth of new blood vessels is an essential part of successful wound healing [14, 15]. Heparin was added to the scaffolds to facilitate sequestering of these growth factors to the collagen scaffold. The interaction between heparin and heparin-binding growth factors (i.e. FGF2 and VEGF) is non-covalent; based on hydrogen bonds and electrostatic interactions that occur between charged groups on the saccharides and specific amino acids in the heparin-binding growth factors [16]. These interactions help to protect growth factors from rapid degradation and diffusion in the wound environment, which prolongs their bioactivity [17].The biological activity of exogenous FGF2 and VEGF is likely confined to the early phases of wound healing, as diffusion and degradation of these factors will start upon scaffold implantation [9]. FGF2 and VEGF may thus have initiated a cascade of events *in utero* that lead to enhanced postnatal regeneration.

Considering the hypothesis that FGF2 and VEGF influence wound healing in an early stage, it stands to reason that their effects are also distinguishable during *in utero* skin closure. This statement was supported by *in vivo* analysis of COL-HEP/GF scaffolds during the prenatal stage. Two weeks post implantation, the wounds treated with a COL-HEP/GF scaffold already looked markedly different compared with collagen-only scaffolds [8]: they were significantly less contracted, the scaffold contained less alpha smooth muscle actin (αSMA)-positive cells (i.e. myofibroblasts), the new epidermis was hyperplastic and there seemed to be less *de novo* extracellular matrix (ECM) deposition at the epidermis-scaffold interface. These observations are classically associated with decreased scarring. However, they are also a downstream result of changes in gene expression, and it is difficult to distill the processes that are involved in skin regeneration only from (immune)histological analysis.

Despite a multitude of studies reporting on materials that improve wound healing, there is still no consensus on which exact biological processes should be targeted to achieve regeneration. Identifying fundamental fetal regeneration-inducing processes would benefit the field of skin repair and the development of regenerative biomaterials for the treatment of spina bifida. Here, we take a retrospective approach by taking a collagen-based biomaterial with proven regenerative capabilities and identify the pivotal processes that it induces based on comprehensive gene expression analysis. Gene expression analysis was performed on fetal full thickness skin wounds two weeks post implantation of scaffolds and biological processes affected by COL-HEP/GF scaffolds were identified.

## Materials and methods

### Production and implantation of collagen scaffolds

Tissue samples from a previously executed study were used for gene expression analysis and histological validation. The full experimental procedures relating to the fetal sheep model are described in the research article published by Hosper et al. [8]. Here a short summary of the experiment is provided.

Porous fibrillar type I collagen scaffolds were prepared and crosslinked using 1-ethyl-3-(3-methyl aminopropyl) carbodiimide (EDC) and N-hydroxysuccinimide (NHS), without and with heparin in the solution. Ø 12 mm collagen-heparin scaffolds were disinfected with 70% ethanol and loaded with fibroblast growth factor 2 (FGF2; human recombinant, R&D systems, Minneapolis, MN, USA) and vascular endothelial growth factor (VEGF; human recombinant, R&D systems) in phosphate buffered saline (PBS).

Three full thickness skin defects (Ø 12 mm) were made on the backs of Dutch-Texel breed fetuses on day 79 of gestation. A plain crosslinked collagen scaffold (COL), or a collagen scaffold crosslinked with heparin (HEP) and loaded with FGF2 and VEGF (COL-HEP/GF) scaffold was implanted in the lesion. The third lesion was left untreated (UNTR). Scaffolds were sutured into place and the wound edges were marked on four sides with a separate non-resorbable suture. At two weeks post-surgery (93 days’ gestation) animals were sacrificed and the wounds of four lambs were evaluated (n = 4). Samples were taken from the defects including surrounding skin and subcutaneous tissue. Normal skin was removed and taken along as a control (NL). These samples were either fixed with 4% formalin in PBS followed by paraffin embedding or snap frozen in liquid nitrogen followed by embedding in O.C.T. Tissue Tek.

### Gene expression analysis

#### Laser capture microscopy

The epidermis and dermis were captured separately using laser capture microscopy as described [9]. Polyethylene naphthalate (PEN) membrane slides (2 μm; Micro-Dissect GmbH, Heborn, Germany) were UV-treated for 30 min at 125 kJ. The cryo-embedded samples were cut with a thickness of 10 μm, fixed onto the UV-treated slides and stored at − 80 °C. The tissue was stained the next day with a fast hematoxylin stain [9, 18]. Epidermis (± 1.5 × 10^6^ μm^2^ per sample) and dermis (± 4 × 10^6^ μm^2^ per sample) were isolated using a Leica Laser dissection microscope (Leica, Eindhoven, The Netherlands), collected in Eppendorf tubes (Eppendorf, Nijmegen, The Netherlands) and stored at − 80 °C before RNA isolation. Invaginated skin appendages were not isolated separately but instead remained in the dermis (Supplementary Information Figure S1).

#### RNA isolation and amplification, cDNA synthesis, labeling, and fragmentation

RNA was isolated used the RNeasy Micro Kit from Qiagen with DNase I treatment on the column according to the manufacturer’s instructions (Qiagen, Hilden, Germany). RNA was eluted in 12 μl RNase-free water (Qiagen). Purity and quality of the RNA samples were determined using the Agilent RNA 6000 Pico kit and Agilent 2100 bioanalyzer (Agilent technologies, Santa Clara, CA, USA). Only samples with an RNA integrity number (RIN) ≥ 6 were used for amplification (average RIN score = 7.5, Supplementary Information Table S1). RNA was amplified (minimum 500 pg) and cDNA was synthesized via Single Primer Isothermal Amplification (SPIA) using the Ovation® Pico WTA System V2 kit (NuGEN Technologies, San Carlos, CA, USA). Afterwards samples were purified using the QIAquick PCR purification kit (Qiagen) according to the instructions of the Ovation® kit. Purified samples were eluted in 20 μl RNase-free water followed by measuring of total cDNA concentration and purity using a NanoDrop 2000 UV–Vis Spectrophotometer (Nanodrop technologies, Wilmington, DE, USA) at 260 and 280 nm. cDNA was labeled and fragmented using the Encore® biotin module (NuGEN) according to the manufacturer’s instructions, whereby the volumes were scaled down with a factor of 0.75 as described previously [9]. In short, 3.4 μg cDNA was fragmented in an end volume of 18.75 μl by incubating for 30 min at 37 °C and 2 min at 95 °C. The fragmented cDNA was labeled with biotin for 60 min at 37 °C and 10 min at 70 °C. Fragmentation was assessed via gel electrophoresis using a 2% agarose gel and determined sufficient if cDNA products were 200 base pairs or smaller. Samples were stored at − 20 °C until loading to microarrays.

#### Microarray gene expression analysis

Gene expression profiles were determined using Ovine Gene 1.0 ST Arrays (Affymetrix, Santa Clara, CA, USA). Fragmented and labeled cDNA was hybridized to the arrays according to the manufacturer’s instructions. Following overnight hybridization (45 °C and 0.33 × g) the arrays were washed and stained using the Affymetrix GeneChip Fluidics Station 450 (Affymetrix). The arrays were scanned using the Affymetrix GeneChip scanner 3000 7G (Affymetrix). Gene expression profiles were extracted from the resulting.CEL files and Partek Genomcis Suite v6.6 (Partek, St. Louis, MO, USA) was used for quality control and normalization. Comparisons were made in each skin layer (‘control condition’ vs. ‘condition of interest’) and differentially expressed genes between groups were identified by performing a one-way analysis of variance (ANOVA) and correction for multiple testing using the Benjamini–Hochberg method. The fold changes were reversed to identify genes that were up or downregulated in the ‘condition of interest’ vs. ‘control condition’: UNTR vs. NL, COL vs. NL, COL-HEP/GF vs. NL, COL vs. UNTR, COL-HEP/GF vs. UNTR, and COL-HEP/GF vs. COL. Lists of differentially expressed genes (DEGs) were obtained by filtering out genes with − 2 > fold change < 2 and *p*-value > 0.05. Transcripts without an annotation that passed the selection threshold were matched to the OviGene master transcript list (OviGene-1_0-st-v1.na36.oar2.tra, Affymetrix) and annotated if possible. Gene transcripts that remained without annotation were excluded from further analysis: across all comparisons an average of 15 ± 4% gene transcripts remained unannotated.

#### Identification of enriched biological processes using DAVID and cluster analysis

The gene ontology (GO) terms enriched in each comparison were obtained using DAVID Bioinformatic Resources (version: v2022q4) [19, 20]. The lists of differentially expressed genes were uploaded to DAVID and converted to human orthologs as the sheep genome is not well-annotated [9, 21, 22]. Unknown gene symbols were updated, if possible, using the NCBI gene browser for *Ovis aries*. The converted gene lists were analyzed in DAVID for enriched GO terms in the category Biological Process (GO_BP_Direct). GO terms with a Bonferroni *p*-value < 0.05 were further analyzed and similar terms were identified and grouped using QuickGO. If possible, similar terms were grouped together under a parent term.

In addition, protein–protein interaction networks were constructed using the DEGs of a comparison. The DEGs were uploaded to String (version 11.5) and the minimum required interaction score was set to 0.7 (high confidence). The networks were exported to Cytoscape (version 3.10.0) for visualization and further analysis. Clusters were identified using the Cytocluster app and ClusterONE algorithm [23, 24]. The resulting clusters (*p* < 0.05) were annotated with biological processes in Enrichr: the term with the lowest adjusted *p*-value was selected [25–27].

### Validation of microarray results

#### Quantitative polymerase chain reaction (qPCR)

The top 10 upregulated genes in the epidermis and dermis of UNTR vs. NL, COL vs. NL and COL-HEP/GF were retrieved. Genes that were upregulated amongst all comparisons within the same skin layer were selected for validation: tenascin C (TNC; dermis), matrix metalloproteinase 13 (MMP13; epidermis), fatty acid binding protein 4 (FABP4; epidermis and dermis). Primer sets, including a reference gene (ribosomal protein L37, RPL37) were obtained from Biolegio (Nijmegen, The Netherlands). Details of the primer sets are presented in Supplementary Information Table S2. Primer sequences were run through NCBI Primer Blast to confirm specificity for the sheep genome (*Ovis aries*) and the primer sets were tested for efficiency (r^2^ > 0.98, slope between − 3.2 and − 3.6 and efficiency 95–105%). qPCR was performed with 2 ng cDNA and iQ™ SYBR® Green Supermix (Bio-Rad, Hercules, CA, USA) using a CFX96 Touch Deep Well™ Real-Time PCR detection system (Bio-Rad) that ran the following program: initial denaturation for 3 min at 95 °C, 40 cycles of 15 s denaturation at 95 °C and 30 s annealing/extending at 60 °C followed by plate reading and a melt curve analysis (65–95 °C). Data were analyzed with CFX Maestro 1.1 software (Bio-Rad) and fold changes (2^−ΔΔCt^) were calculated using Microsoft® Excel.

#### Immunohistochemistry

Tissue sections with a thickness of 5 μm were cut from paraffin embedded samples. The sections were deparaffinized with xylene and decreasing concentrations of ethanol. First, endogenous peroxidase activity was blocked by incubating the sections for 30 min with 3% H_2_O_2_ in methanol followed by washing 3 × 5 min with Tris buffered saline (TBS: 50 mM Tris, 0.9% NaCl, pH 7.4). The sections were blocked for 15 min in 1% bovine serum albumin (BSA) dissolved in phosphate buffered saline (PBS: pH 7.2). Sections were incubated for 45 min with an antibody raised against human adipocyte FABP4 (1:100 in 1% BSA-PBS, [28]) and then washed 3 × 5 min in TBS. Thereafter the sections were incubated for 45 min with peroxidase conjugated goat anti-rabbit IgG (1:5000 in 1% BSA-PBS, Pierce Biotechnology, Rockford, IL, USA). Following 3 × 5 min washing in TBS, the staining was visualized with 3,3′-diaminobenzidine tetrahydrochloride (DAB). The sections were counterstained with hematoxylin before mounting and imaged with brightfield microscopy.

## Results

### Treatment with COL-HEP/GF leads to an increase in differentially expressed genes

Gene expression analysis of the epidermis and dermis at two weeks post wounding revealed numerous genes that were differentially expressed between the various treatments (Table 1, Dataset S1 and Dataset S2). Between UNTR wounds and normal skin (UNTR vs. NL) 578 differentially expressed genes (DEGs) were found in the epidermis and 415 DEGs were found in the dermis. Treatment of wounds with COL or COL-HEP/GF led to an increase in DEGs when compared to NL. In COL vs. NL there were 962 DEGs in the epidermis and 1607 in the dermis. The number of DEGs increased even more in COL-HEP/GF vs. NL, with 1168 DEGs in the epidermis and 1520 DEGs in the dermis. When wounds treated with COL or COL-HEP/GF were compared to UNTR wounds the number of DEGs was smaller, indicating the gene expression profiles were more similar between these conditions. In COL vs. UNTR only 197 DEGs were found in the epidermis compared to 602 DEGs in the dermis. These numbers were comparable to COL-HEP/GF vs. UNTR, where 362 and 627 DEGs were identified in the epidermis and dermis, respectively. A comparison of COL-HEP/GF vs. COL revealed 286 DEGs in the epidermis and 618 DEGs in the dermis. This indicates there is a change in the gene expression profile when wounds were treated with the functionalized collagen scaffold.

**Table 1:** Number of differentially expressed genes (DEGs) in the epidermis and dermis at 2 weeks post-implantation

	UNTR vs. NL	COL vs. NL	COL-HEP/GF vs. NL	COL vs. UNTR	COL-HEP/GF vs. UNTR	COL-HEP/GF vs. COL
Epidermis						
Total	578	962	1168	197	362	286
Upregulated (%)	355 (61)	496 (52)	776 (66)	87 (44)	250 (69)	229 (80)
Downregulated (%)	223 (39)	466 (48)	392 (34)	110 (56)	112 (31)	57 (20)
Dermis						
Total	415	1607	1520	602	627	618
Upregulated (%)	180 (43)	629 (39)	938 (62)	250 (42)	445 (71)	460 (74)
Downregulated (%)	235 (57)	978 (61)	582 (38)	352 (58)	182 (29)	158 (26)

The table displays the total number of DEGs identified in each comparison (*p* < 0.05 and − 2 > fold change < 2), and the number of genes that were up or downregulated in the ‘condition of interest’ compared to a control

NL, normal skin; UNTR, untreated wound; COL, bare collagen scaffold; COL-HEP/GF, collagen-heparin scaffold functionalized with the growth factors FGF2 and VEGF

**Fig. 1:**
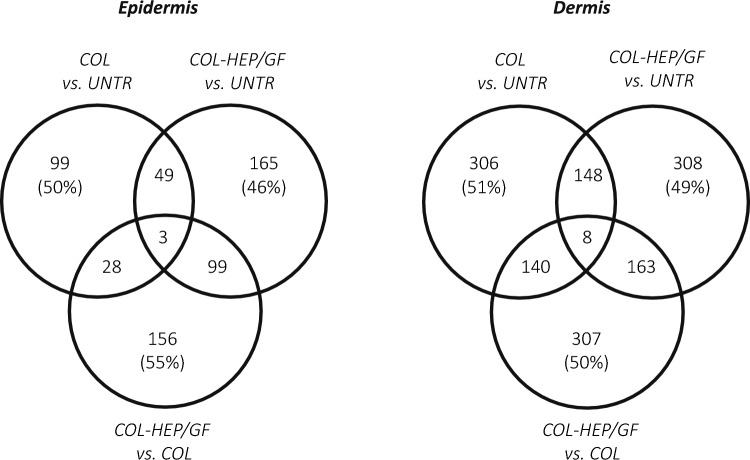
Numbers of shared and unique differentially expressed genes (DEGs) in the epidermis and dermis at 2 weeks post-implantation. The numbers in overlapping regions represent the shared DEGs between comparisons. The numbers in non-overlapping regions are the unique DEGs in each comparison, with the percentage of unique to total DEGs in this comparison. UNTR, untreated wound; COL, bare collagen scaffold; COL-HEP/GF, collagen-heparin scaffold functionalized with the growth factors FGF2 and VEGF

The difference between the treatments was further evaluated by comparing COL vs. UNTR, COL-HEP/GF vs. UNTR and COL-HEP/GF vs. COL for unique and overlapping DEGs (Fig. 1). The overlapping DEGs indicate that the wound healing responses to the bare and functionalized collagen scaffold is in part similar. On average 50 ± 3% of DEGs was unique to each comparison, including in COL-HEP/GF vs. COL. There was no large difference in the percentage of unique genes found in the epidermal and dermal comparisons, even though the overall number of DEGs was higher in the dermis compared to the epidermis. The unique DEGs identified in COL-HEP/GF vs. COL represent the subset of genes that are especially affected by the functionalized collagen scaffold and these may be involved in the processes that led to the pronounced wound healing effects reported by Hosper et al*.* and Oostendorp et al*.* [8, 9].

### Validation of microarray data through qPCR and immunohistochemistry

Three genes that were among the top-10 upregulated genes (Supplementary Information Table 3) in the microarrays of UNTR vs. NL, COL vs. NL and COL-HEP/GF vs. NL were selected for validation through qPCR: matrix metalloproteinase 13 (MMP13, epidermis), tenascin C (TNC, dermis) and fatty acid binding protein 4 (FABP4, epidermis and dermis). Data are presented in Supplementary Information Figure S2. MMP13 expression could be detected in all sheep for UNTR, COL and COL-HEP/GF samples and in one normal skin sample (sheep 2) (Supplementary Information Figure S2A). In all other NL samples, the expression of MMP13 was below detection. This indicates that MMP13 expression (2^−ΔCt^) is increased in the wound-derived samples (UNTR, COL, COL-HEP/GF) compared to the expression in NL. The expression of TNC was also found to be upregulated in all samples of the dermis according to qPCR (Supplementary Information Figure S2B). FABP4 was upregulated in all samples of the epidermis with the exception for sheep 4, where an expression level of − 2.2 was seen for UNTR vs. NL (Supplementary Information Figure S2C). In the dermis variation in FABP4 expression between individual sheep was observed (Supplementary Information figure S4D). All wound-derived samples of sheep 2 and sheep 3 showed an increase in FABP4 expression compared to normal skin. In sheep 1 and sheep 4 both UNTR samples had a lower expression level compared to normal skin. In sheep 4 the FABP4 expression of the COL sample was somewhat lower than normal skin. It is important to note is that in the microarray dataset FABP4 was not amongst the DEGs of UNTR vs. NL, which may explain the inconsistent results found with qPCR. Aside from some deviations the gene expression values obtained through qPCR matched those obtained with the microarray.

The microarray data were further supported through immunohistochemistry on tissue sections with an antibody specific to the FABP4 (Supplementary Information Figure S3). In normal skin (NL) the staining of FABP was relatively weak and limited to the epidermis, with no staining observed in the dermis. In comparison, the epidermis of UNTR samples displayed a strong signal for FABP4 in the epidermis, with no stain in the dermis. In COL-treated samples, the FABP4 signal was again present throughout the entire epidermis. The same was observed for COL-HEP/GF samples; with the epidermis staining positive for FABP4. Remnants of collagen scaffolds in the dermal layer (COL and COL-HEP/GF) were infiltrated with cells. The cells, but also the scaffolds, stained positive for FABP4. As FABP4 is a cytosolic protein, this effect may be partially due to non-specific staining of the collagen fibers. In summary, the immunohistochemistry results largely correspond to the data obtained from the microarrays.

### Treatment with COL-HEP/GF leads to an increase in enriched biological processes.

A screen for enriched biological processes amongst the DEGs in the epidermis and dermis was conducted using DAVID bioinformatics resources. Lists of enriched biological processes (*p* < 0.05) were generated for each comparison (Supplementary Data S1 and S2) and the gene ontology (GO) terms were grouped together using QuickGO. The biological process identified in the epidermis and dermis of COL vs. UNTR, COL-HEP/GF vs. UNTR and COL-HEP/GF vs. COL are presented in Table 2.

**Table 2:** Biological processes enriched at 2 weeks post wounding (Bonferroni *p* < 0.05)

Epidermis	FE	Dermis	FE
COL vs. UNTR			
No enriched terms		No enriched terms	
COL-HEP/GF vs. UNTR			
GO:0042060 ~ wound healing	6.35	GO:0045071 ~ negative regulation of viral genome replication	8.29
GO:0051607 ~ defense response to virus	4.07	GO:0009615 ~ response to virus	7.49
GO:0010628 ~ positive regulation of gene expression	2.83	GO:0051607 ~ defense response to virus	5.03
		GO:1,902,533 ~ positive regulation of intracellular signal transduction	
		GO:1,901,224 ~ positive regulation of NIK/NF-kappaB signaling	5.94
		GO:0070374 ~ positive regulation of ERK1 and ERK2 cascade	3.33
		GO:0006935 ~ chemotaxis	4.51
		GO:0030335 ~ positive regulation of cell migration	3.94
		GO:0006954 ~ inflammatory response	3.52
		GO:0001525 ~ angiogenesis	3.42
		GO:0006915 ~ apoptotic process	2.63
		GO:0006955 ~ immune response	2.46
		GO:0045087 ~ innate immune response	2.58
		GO:0007155 ~ cell adhesion	2.25
COL-HEP/GF vs. COL			
GO:0009615 ~ response to virus	6.94	No enriched terms	
GO:0051607 ~ defense response to virus	5.60		

Enriched terms were identified using DAVID bioinformatics resources and terms without an FE value were grouped under a parent term that was separately identified using QuickGO

FE, fold enrichment; GO. gene ontology; UNTR, untreated wound; COL, bare collagen scaffold; COL-HEP/GF, collagen-heparin scaffold functionalized with the growth factors FGF2 and VEGF

In COL vs. UNTR no enriched biological processes were found amongst the 64 total terms in the epidermis and 139 total terms in the dermis. Multiple GO terms were enriched in COL-HEP/GF vs. UNTR: 3 out of 153 terms and 13 out of 356 terms were enriched in the epidermis and dermis, respectively. In both layers ‘defense response to virus’ was enriched, along with the dermis-specific terms: ‘negative regulation of viral genome replication’, ‘immune response’ and ‘inflammatory response’. This may indicate that the COL-HEP/GF scaffold induces some reaction of the immune system, which was not the case for COL-only scaffolds. No obvious signs of immune system activation in response to the COL-HEP/GF scaffolds were reported by Hosper and associates [8] but it should be noted that the exact onset of fetal immunocompetency is elusive [29]. The other enriched terms indicate COL-HEP/GF scaffolds influence various aspects of the healing response. ‘Wound healing’ was enriched in the epidermis and in the dermis ‘positive regulation of cell migration’, ‘angiogenesis’, ‘apoptotic response’ and ‘cell adhesion’ were enriched. Additionally, the enrichment of ‘positive regulation of gene expression’ (epidermis), ‘positive regulation of intracellular signal transduction’ (dermis) and ‘chemotaxis’ (dermis) in COL-HEP/GF vs. UNTR could indicate that the presence of FGF2, VEGF and heparin increases cellular activity. In COL-HEP/GF vs. COL only two out of a 106 total terms were enriched the epidermis. These were: ‘defense response to virus’ and its parent term ‘response to virus’. No enriched terms were found in the dermis (117 total terms). The lack of enriched biological processes is curious given the macroscopic and microscopic differences between COL and COL-HEP/GF defects two weeks after implantation that were previously described by Hosper et al. [8].

The biological processes enriched in COL-HEP/GF vs. UNTR do indicate the functionalized collagen scaffold influences the wound healing response. This observation is supported by the biological processes that were enriched when the wounded conditions were compared to normal skin (‘NL’). COL-HEP/GF scaffolds led to a noticeable increase in enriched terms compared to COL scaffolds. Moreover, most of the terms found in COL vs. NL were also present COL-HEP/GF vs. NL. In the epidermis of COL vs. NL, a total of five terms were enriched and four of these terms were also found amongst the 25 enriched terms in the epidermis of COL-HEP/GF vs. NL. The same was true for the dermis: nine terms were enriched in COL vs. NL and eight of these terms were also found amongst the 60 enriched terms in COL-HEP/GF. The unique enriched terms in COL-HEP/GF vs. NL encompassed a multitude of processes. Such as ‘endodermal and epithelial cell differentiation’, ‘extracellular matrix organization’ and ‘epithelial to mesenchymal transition’. Several other unique terms were found: ‘aging’, ‘odontogenesis of dentin-containing tooth’, ‘ossification’, ‘kidney development’, ‘skeletal system development’ and ‘heart development’. These terms may seem out of place, but the genes grouped under these biological processes often have functions in multiple tissues and DAVID does not consider the tissue origin of a dataset. For example, many genes belonging to the terms ‘aging’ and ‘ossification’ are also known to play a role in wound healing: lysyl oxidase-like 2 (LOXL2), TIMP metallopeptidase inhibitor 1 (TIMP1), matrix metalloproteinase 2 (MMP2), and transforming growth factor beta 1 (TGFB1). In order to find out which processes are different in wounds treated with COL-HEP/GF compared to COL the DEGs were further analyzed for the presence of gene clusters.

### Identifying the differences between COL-HEP/GF and COL from clustered differentially expressed genes

To find out which biological processes the DEGs of COL-HEP/GF vs. COL belong to, a protein–protein interaction network was constructed. This interaction network was clustered and each cluster was annotated with a GO-term (Table 3, Supplementary Information Figures S4 and S5).

**Table 3:** Clusters of DEGs (differentially expressed genes) in the epidermis and dermis of COL-HEP/GF vs. UNTR. annotated with gene ontology (GO) terms for biological processes

#	GO term	Gene symbol (FC)	Nodes, density, quality
COL-HEP/GF vs. COL—Epidermis			
1	Defense Response To Virus (GO:0051607)	USP18 (9.82), IFIT1 (8.76), IFIT2 (5.02), MX2 (4.84), PARP14 (4.60), CMPK2 (4.46), IFIH1 (3.21), HERC5 (3.16), IRF9 (3.11), SAMD9 (3.08), STAT1 (2.94), HERC6 (2.39), UBA7 (2.25), ADAR (2.21), PLSCR1 (-1.08)	19, 0.57, 0.92
2	Vesicle-Mediated Transport (GO:0016192)	AP1M1 (2.49), ADRB2 (2.32), WNT5A (2.30), UBQLN2 (2.25), AP2B1 (2.18), EPN3 (-2.19), PANK1 (-2.28)	7, 0.33, 1.00
3	Regulation Of Telomere Maintenance (GO:0032204)	NMI (5.07), RUNX1 (3.82), CCND2 (3.19), MYC (2.72), MOV10 (2.27), AKT3 (2.26), PML (2.23), HBP1 (2.21), PRAM1 (-2.82)	9, 0.31, 0.79
4	Positive Regulation Of Cellular Process (GO:0048522)	PDGFRA (5.34), VEGFC (4.55), FCGR2B (4.46), CDH11 (4.00), PDGFRB (3.45), ITGAX (2.71), SHC1 (2.03), CD3E (-2.05)	8, 0.43, 0.75
5	COPII-coated Vesicle Cargo Loading (GO:0090110)	ARFGAP3 (2.80), SEC23A (2.77), KDELR1 (2.63), SEC31A (2.44), SURF4 (2.20), SEC24B (2.07)	6, 0.47, 1.00
6	Positive Regulation Of Collagen Biosynthetic Process (GO:0032967)	BGN (5.18), COL6A2 (3.71), FBN1 (3.35), MMP2 (3.26), TGFB1 (2.69), PLOD1 (2.49), CD81 (2.25), VIM (2.20), TPM1 (2.02)	9, 0.22, 0.73
COL-HEP/GF vs. COL—Dermis			
1	Positive Regulation Of Mitotic Sister Chromatid Separation (GO:1,901,970)	BIRC5 (3.15), KPNA2 (2.84), ANLN (2.71), CDCA8 (2.7), ASF1B (2.59), ANAPC11 (2.57), CCNB2 (2.54), MCM7 (2.34), DIAPH3 (2.34), RAD51AP1 (2.28), PIAS3 (2.24), STIL (2.22), MCM6 (2.18), LMNB1 (2.13), NUSAP1 (2.10), KIF18A (2.08), DLGAP5 (2.06), CDC20 (2.06), DNAJC9 (2.05), CKAP2L (2.04), HDAC6 (2.03), MKI67 (2.02), BUB1B (2.01), RORA (-2.73)	24, 0.37, 0.87
2	Protein Targeting To Mitochondrion (GO:0006626)	DLAT (4.67), TMEM11 (3.81), PDHA1 (3.42), TOMM6 (2.89), LYRM4 (2.82), CS (2.61), GRPEL1 (2.55), TIMM22 (2.48), DNAJC19 (2.39), HSPA9 (2.34), TOMM40L (2.23), SDHA (2.14), SAMM50 (2.09)	14, 0.31, 1.00
3	RNA Splicing, Via Transesterification Reactions With Bulged Adenosine As Nucleophile (GO:0000377)	SNRPD3 (3.44), AHSA2 (3.00), SF3B4 (2.82), CSTF1 (2.58), TRNAU1AP (2.35), SNRPA (2.25), SF3A1 (2.23), PPIG (2.14), SRSF1 (2.06), SNRNP27 (2.05), RNPS1 (2.03)	13, 0.35, 0.93
4	Defense Response To Virus (GO:0051607)	IFI27 (15.77), IFIT2 (6.81), USP18 (6.64), DDX58 (5.33), PARP12 (5.07), RNF213 (4.88), RSAD2 (4.81), PARP14 (4.11), EIF2AK2 (3.74), CMPK2 (3.15), HERC6 (3.14), TRIM21 (2.86), TRIM25 (2.73), IFITM1 (2.48)	14, 0.34, 0.80
5	Translation (GO:0006412)	MRPL30 (3.89), MRPL10 (2.86), MRPS18B (2.76), MRPL40 (2.52), MRPS30 (2.32), MRPS14 (2.26), MRRF (-2.10), MRPL22 (-2.48)	8, 1.00, 1.00
6	RNA Splicing, Via Transesterification Reactions With Bulged Adenosine As Nucleophile (GO:0000377)	SNRPD3 (3.44), SF3B4 (2.82), CSTF1 (2.58), TRNAU1AP (2.35), SNRPA (2.25), SF3A1 (2.23), SRSF1 (2.06), RNPS1 (2.03), U2AF2 (2.02)	9, 0.64, 0.82
7	Regulation Of DNA Damage Response, Signal Transduction By P53 Class Mediator (GO:0043516)	PSMB9 (3.69), PSMB10 (3.47), SPRED2 (2.99), PSMB1 (2.36), PSMD10 (2.18), PSME2 (2.08), PRICKLE1 (-2.41)	7, 0.95, 0.87
8	Cellular Response To Interleukin-2 (GO:0071352)	IL2RB (3.70), IL2RG (3.51), NMI (3.31), IL7R (2.84), CD247 (2.66), MAP2K2 (2.31), PLEKHO1 (2.07), PPP2R2B (-3.62)	8, 0.39, 0.79
9	Response To Reactive Oxygen Species (GO:0000302)	HMOX1 (3.88), ESD (3.12), ACP1 (2.70), PGM2 (2.39), GSTP1 (2.26), DNPEP (2.09), GPX7 (-4.71)	7, 0.43, 0.82
10	Regulation Of Type II Interferon Production (GO:0032649)	TNFSF10 (7.37), ZBP1 (7.36), CFLAR (2.21), TLR3 (2.12), TICAM1 (2.10), RIPK3 (2.08), IL1R1 (-3.25)	7, 0.43, 0.82
11	Collagen Fibril Organization (GO:0030199)	MOV10 (2.24), COL1A1 (-2.01), CBFB (-2.13), ITGA11 (-2.37), COL1A2 (-2.42), COL11A1 (-4.61), ITGBL1 (-9.93)	7, 0.38, 0.73
12	TOR Signaling (GO:0031929)	EIF4EBP2 (3.92), AP1M1 (3.11), MET (2.34), NRG4 (-2.04), RPS6KB2 (-2.13), PGF (-2.41), IRS1 (-2.48), EREG (-2.72)	8, 0.25, 0.70
13	Response To Reactive Oxygen Species (GO:0000302)	HMOX1 (3.88), UBL4A (3.26), GSTP1 (2.26), STX5 (-2.45), TRAPPC3 (-2.72), GPX7 (-4.71)	6, 0.40, 0.86
14	Negative Regulation Of Fibroblast Growth Factor Receptor Signaling Pathway (GO:0040037)	ETV4 (7.58), VHL (6.08), SPRY2 (4.68), ETV5 (4.07), SPRY4 (2.74), SPRY1 (2.66)	6, 0.40, 0.86
15	Ceramide Metabolic Process (GO:0006672)	SDS (3.22), ORMDL2 (2.34), SPTLC2 (2.24), ORMDL1 (-6.1), SPTSSB (-6.38)	4, 0.60, 1.00
16	Regulation Of Transcription Elongation By RNA Polymerase II (GO:0034243)	ELL (2.76), SSRP1 (2.58), CDK12 (2.30), CCNT1 (2.22), HEXIM1 (2.02), NELFE (2.02), TCEA1 (1.17), SUPT4H1 (-2.70)	8, 0.64, 0.62
17	Collagen Fibril Organization (GO:0030199)	KPNA2 (2.84), MOV10 (2.24), APOBEC3B (2.02), COL1A1 (-2.01), CBFB (-2.13), ITGA11 (-2.37), COL1A2 (-2.42), COL11A1 (-4.61), ITGBL1 (-9.93)	9, 0.28, 0.56
18	rRNA Processing (GO:0006364)	ZNRD1 (2.76), EXOSC1 (2.68), LYAR (2.64), MAK16 (2.52), DDX10 (-1.16)	5, 0.50, 0.88
19	Transmembrane Receptor Protein Tyrosine Kinase Signaling Pathway (GO:0007169)	NGEF (2.49), EPHA3 (2.43), FGF18 (-2.63), NCAM1 (-3.29), FGFR2 (-3.32)	5, 0.40, 0.67
20	Negative Regulation Of Peptidase Activity (GO:0010466)	RETN (5.27), RBP4 (3.4), CFD (2.99), SERPINE1 (2.31), SERPINA12 (-4.70)	5, 0.50, 0.71
21	Ephrin Receptor Signaling Pathway (GO:0048013)	NT5E (3.37), ANPEP (2.84), NGEF (2.49), EPHA3 (2.43), CDO1 (-3.16), NCAM1 (-3.29)	6, 0.33, 0.63
22	Positive Regulation Of Mononuclear Cell Migration (GO:0071677)	CXCL10 (17.48), CXCL3 (4.71), ACKR4 (3.95), CCR1 (2.55), CCR7 (2.43), CCR8 (2.00)	6, 0.53, 0.62
23	Regulation Of Axon Guidance (GO:1,902,667)	TUBB2B (2.28), TCP11L1 (2.11), KIF18A (2.08), KIAA1279 (2.03), TBCA (2.01), CCDC23 (-3.80)	6, 0.33, 0.36
24	High-Density Lipoprotein Particle Remodeling (GO:0034375)	MT2A (17.57), APOD (5.18), APOA1 (2.6), SCARB1 (2.45), LIPG (-3.30),	5, 0.40, 0.67

Clusters were identified in protein–protein interaction networks using the ClusterONE application (*p* < 0.05) and each cluster was matched to a biological process using Enrichr. The DEGs in each cluster are identified by their gene symbol with the corresponding fold change (FC)

Nodes, number of nodes in a particular cluster; density, average edge weight of a cluster; quality, ratio of edge weights inside a cluster over total edge weights (inside and outside) of a cluster

Several clusters were annotated with terms that are associated with the immune system. In both skin layers a cluster was found that was annotated with the term ‘defense response to virus’, which matches the results obtained with DAVID. Dermal cluster 22 contained genes related to ‘positive regulation of mononuclear cell migration’. This correlates with the ‘cellular response to interleukin 2’ (dermal cluster 8): interleukin 2 is a cytokine produced primarily by T cells (a mononuclear cell) and it primarily activates lymphocytes that are important during viral infections [30]. ‘Regulation of type II interferon production’ (dermal cluster 10) indicates that the adaptive immune cells such as T cells which produce interferon gamma—the only known type II interferon—are affected [31]. The majority of genes in these clusters were upregulated and taken together it seems that COL-HEP/GF activate a strong cytokine response.

COL-HEP/GF scaffolds induced a general cellular response (‘positive regulation of cellular process’, epidermal cluster 4) and various clusters were annotated with terms related to increased cell proliferation and activity. Cluster 12 in the dermis was annotated with ‘TOR signaling’ and this intracellular cascade is implicated in the build-up of cell mass [32]. Three clusters were associated with cell division: ‘regulation of telomere maintenance’ (epidermal cluster 3), ‘positive regulation of mitotic sister chromatid separation’ (dermal cluster 1) and ‘regulation of DNA damage response’ (dermal cluster 7). Dermal cluster 23 was annotated with ‘axon guidance’ but the DEGs within this cluster were associated with microtubules. Part of the cytoskeleton, microtubules are important in a variety of cellular processes by facilitating intracellular transport (vesicles) and cell division [33]. Gene transcription was also affected in COL-HEP/GF-treated wounds: dermal clusters 3 and 6 shared a term for RNA splicing (‘RNA splicing, via transesterification reactions with bulged adenosine as nucleophile’) and dermal cluster 16 was annotated for ‘regulation of transcription elongation by RNA polymerase II’.

There was evidence of increased protein production in COL-HEP/GF compared to COL: ‘translation’ (dermal cluster 5) is a general term for the metabolic process during which a protein is formed. Four clusters were associated with the mitochondria, which also indicates increased cell activity: ‘protein targeting to mitochondrion’ (dermal cluster 2), ‘rRNA processing’ (dermal cluster 18) and ‘response to reactive oxygen species’ (dermal clusters 9 and 13). Two clusters in the epidermis were associated with vesicles: ‘vesicle-mediated transport’ (epidermal cluster 2) and ‘COPII-coated vesicle cargo loading’ (epidermal cluster 5). The first term is a general one, but the latter is more specific as COPII-coated vesicles transport their cargo to the Golgi apparatus [34]. This organelle is responsible for protein and lipid processing and packaging. The presence of ‘negative regulation of peptidase activity’ (dermal cluster 20) may indicate protein breakdown is reduced in COL-HEP/GF-treated wounds. Cluster 15 was annotated with ‘ceramide metabolic process’. Ceramides are bioactive sphingolipids that are an integral part of the plasma membrane. Increased cell numbers would require the synthesis of plasma membrane components, but ceramides can also influence intra- and intercellular messaging [35]. The annotation of dermal cluster 24 with ‘high-density lipoprotein (HDL) particle remodeling’ indicates there is a change in the lipid metabolism, on the other hand HDL particles can play a role in cell–cell communication.

**Fig. 2:**
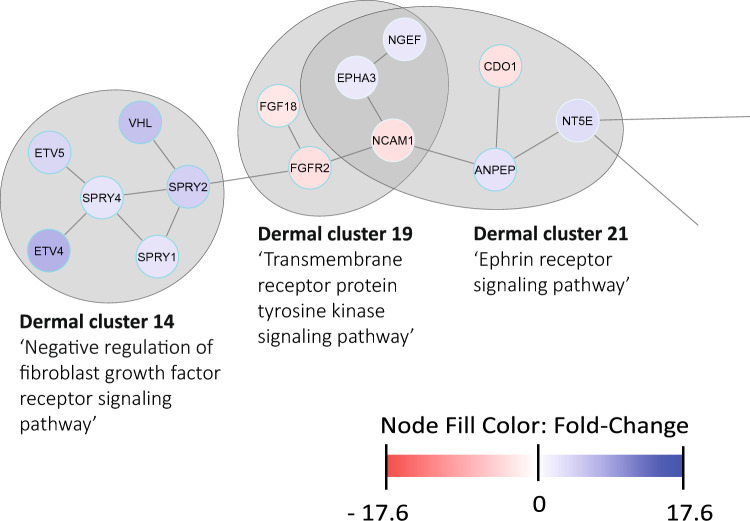
Differentially expressed genes (DEGs) belonging to dermal cluster 14, 19 and 21 indicate a reaction towards the active components of the COL-HEP/GF scaffold compared to COL-only. Every cluster is annotated with a biological process. Each node represents a DEG and grey lines (‘edges’) connect to other DEGs in the network. A bright blue border indicates the DEG is part of one cluster, a light blue border indicates the DEG is part of multiple clusters. Upregulated DEGs are filled with blue, with dark blue to light blue represents the most to least upregulated. Downregulated DEGs are filled with red, where dark red to light red represents the most to least downregulated

Cluster 14 was annotated with the term ‘negative regulation of fibroblasts growth factor receptor signaling pathway’. The DEGs belonging to this cluster—all of which were upregulated in COL-HEP/GF—are known downstream targets of fibroblast growth factor signaling. Amongst these DEGs are members of the ETV-family of transcription factors and the Sprouty family of tyrosine kinase inhibitors, which may signal that the exogenous application of FGF2 results in control of signaling pathways (Fig. 2). A related term was found for cluster 19: ‘transmembrane receptor protein tyrosine kinase signaling pathway’—the fibroblast growth factor receptor family is a member of the receptor tyrosine kinases. This cluster contained five DEGs of which three were downregulated in COL-HEP/GF: fibroblast growth factor 18 (FGF18, its downregulation has been associated with hair follicle regeneration [36]), neural cell adhesion molecule 1 (NCAM1, a ligand for FGF receptors) and fibroblast growth factor receptor 2 (FGFR2). Another term related to receptor tyrosine kinases was assigned to cluster 21: ‘ephrin receptor signaling pathway’. Ephrin receptors are receptor tyrosine kinases which facilitate cell–cell signaling and ephrin signaling pathways influence a multitude of processes ranging from embryonic development to tissue homeostasis.

**Fig. 3:**
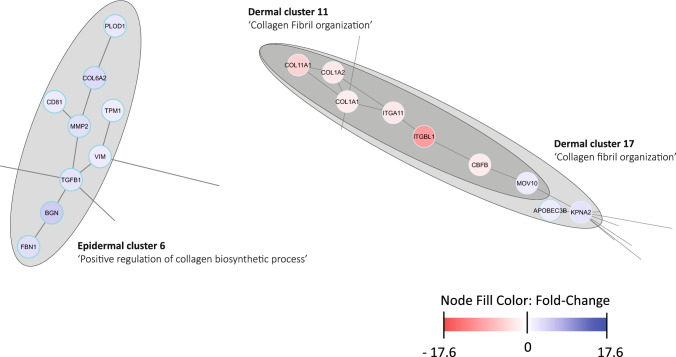
Differentially expressed genes (DEGs) belonging to epidermal cluster 6, and dermal clusters 11 and 17 indicate the role of COL-HEP/GF in extracellular matrix deposition and organisation. Every cluster is annotated with a biological process. Each node represents a DEG and grey lines (‘edges’) connect to other DEGs in the network. A bright blue border indicates the DEG is part of one cluster, a light blue border indicates the DEG is part of multiple clusters. Upregulated DEGs are filled with blue, with dark blue to light blue represents the most to least upregulated. Downregulated DEGs are filled with red, where dark red to light red represents the most to least downregulated

Collagen deposition and organization were impacted by COL-HEP/GF scaffolds: ‘positive regulation of collagen biosynthetic process’ (epidermal cluster 6) and ‘collagen fibril organization’ (dermal cluster 11 and 17). A variety of DEGs were included in these clusters, both up- and down-regulated (Fig. 3). COL6A2 (upregulated, epidermis) is a part of the interconnective tissue at the interface of the basement membrane and is associated organization of the dermal matrix [37]. Matrix metalloproteinase 2 (MMP2, epidermis) was upregulated: this enzyme cleaves type IV collagen, a major component of the basement membrane. COL1A1 and COL1A2, coding for the alpha chains of type I collagen were downregulated (dermis). Fibrotic skin is characterized by an overabundance of this collagen hence its downregulation is a positive sign for skin regeneration [38]. COL11A1 was also downregulated (dermis): type XI collagen regulates type I and type II collagen fibrillogenesis, and it can bind heparin. Various other collagen- and ECM-associated genes were affected by treatment with COL-HEP/GF, such as lysyl hydroxylase 1 (PLOD1), which was upregulated in the epidermal dataset. This enzyme enables the hydroxylation of lysine residues and is essential for collagen fibril maturation [39]. Vimentin (VIM, epidermis) and fibrillin (FBN1, epidermis) were both upregulated in COL-HEP/GF treated wounds. These proteins are important components of the ECM, including the dermal–epidermal junction, and are known to impact wound healing [40–43]. Transforming growth factor beta 1 (TGF-β1) was also upregulated in COL-HEP/GF. This gene is essential during wound healing and regulates many processes, on the other hand its continued over-activity is associated with scarring [44]. The combination of up- and down-regulated DEGs indicates the deposition and organization of the ECM is carefully controlled in wounds treated with COL-HEP/GF.

## Discussion

To promote *in utero* skin generation for the treatment of the severe developmental condition spina bifida, our research group developed a biomaterial composed of a porous type I collagen scaffold, functionalized with heparin, FGF2 and VEGF (COL-HEP/GF). The regenerative potential of COL-HEP/GF scaffolds was investigated using a fetal sheep full thickness wound model. Implantation of the COL-HEP/GF scaffolds resulted in enhanced skin regeneration both pre- and postnatally [8, 9]. At two weeks post implantation, the macroscopic and microscopic differences between the treatments were already quite pronounced. To identify the fetal biological processes underlying these differences we applied gene expression analysis. By unravelling the processes associated with the early phases of skin regeneration novel insights may be obtained for the construction of informative biomaterials.

We performed gene expression analysis of wounds two weeks post implantation to determine which biological processes underlie the improved skin regeneration induced by COL-HEP/GF. First, the differentially expressed genes (DEGs) between treatments were identified and the Database for Annotation, Visualization, and Integrated Discovery (DAVID) was used to extract overrepresented biological processes. COL-HEP/GF scaffolds, when compared to normal skin (NL) or untreated (UNTR) wounds, affected various processes associated with wound healing: ‘extracellular matrix organization’, ‘epithelial to mesenchymal transition’, ‘wound healing’, ‘cell migration’, and ‘angiogenesis’. These processes are also known to be influenced by FGF2 and VEGF [10, 11, 45, 46]. Only two biological processes were enriched in COL-HEP/GF vs. COL: ‘response to virus’ and ‘defense response to virus’. This was unexpected given the macro- and microscopic differences that were already discernable at this timepoint.

In order to delve deeper into the processes influenced by COL-HEP/GF, the DEGs identified between COL-HEP/GF vs. COL were further explored using protein–protein interaction (PPI) networks. PPI networks identify clusters of highly related nodes (i.e. ‘genes’) and these clusters can subsequently be annotated with biological processes [47, 48]. This approach led to the identification of various processes that are affected by COL-HEP/GF scaffolds. A number of these clusters were annotated with terms related to protein translation, chromatid separation and telomere maintenance. This matches with the hyperplastic character of the new skin observed at this timepoint [8], as the rapid build-up of cell mass requires energy, active cell division and protein production. In both skin layers ‘defense response to virus’ was identified, which matched the enrichment analysis performed with DAVID, several other immune system-related clusters were present as well. The inflammatory response is an extensive part of adult wound healing [49, 50] whereas in early-gestation fetal wounds, which regenerate without scarring, there is a distinct absence of inflammation [51, 52]. While wound healing during late gestation results in scar formation, little is known about the contribution of the immune system during this period. Generally, implantation of a foreign material would lead to the foreign body response—where macrophages fuse into foreign body-type giant cells in order to degrade the implant—but the tissue sections of both COL- and COL-HEP/GF-treated prenatal wounds hardly contained any macrophages or giant cells [8]. Another explanation for the effect of COL-HEP/GF on immune-related processes may be related to the production of the cytokines and growth factors to steer cellular processes. The type II interferon (or: ‘IFNγ’) signaling pathway is known for its role in inflammation, but it has also been implicated directly in wound healing [53, 54].

The remaining clusters identified in wounds treated with COL-HEP/GF sketch a picture of a balanced wound environment with controlled intracellular signaling and ECM production. Especially noticeable was a cluster annotated with: ‘negative regulation of fibroblast growth factor receptor signaling pathway’. The FGF2 response is biphasic: a low concentration of FGF2 instigates a stronger cellular response compared to high concentrations of FGF2. This effect is partially related to the oversaturation of FGF receptors, which prevents the correct formation of the signaling unit [55]. Considering the exogenous application of FGF2 via the functionalized collagen scaffold it may be that FGF2 signaling was reduced because of a too high concentration. More evidence of altered signaling pathways was found in clusters annotated with terms that are related to tyrosine kinase receptors and their downstream pathways, as both FGF2 and VEGF signal via tyrosine kinases. In COL-HEP/GF-treated wounds the epidermis and dermis both contained terms for the regulation of collagen biosynthesis and collagen fibril organization, indicating controlled ECM turnover. This matches the results reported by Hosper and associates, where COL-HEP/GF wounds showed less ECM deposition at two weeks post implantation and the scaffold was degraded slower compared to COL-treated wounds [8]. This effect is especially relevant as a main characteristic of fibrotic wound healing is an abundant deposition of ECM proteins—of type I collagen—and preventing this uncontrolled deposition is an important goal in regenerative strategies [38]. Overall, the biological processes that are affected by COL-HEP/GF scaffolds indicate the combination of the pro-wound healing agents FGF2 and VEGF enact a subtle control over cell signaling processes and ECM organization at two weeks post-implantation.

A limitation of this study is the absence of a collagen-heparin scaffold. The effect of heparin on the wound environment should be considered. Heparin and heparan sulfate are known for their regulating and stabilizing effect on growth factors, cytokines and ECM ligands [56]. Supplementation of wounds with heparin or heparan sulfate, either in its natural form or as a mimetic, was previously shown to improve wound healing outcomes [57–62]. The pro-regenerative effects seen in collagen-heparin-FGF2-VEGF scaffolds may thus in part be the result of the stabilizing presence of heparin or the binding of endogenously produced heparin-binding growth factors. It should also be noted that gene expression does not necessarily directly affect protein levels. However, the similarities between the biological processes identified in this study and the observations reported by Hosper and associates support the relevance of the gene expression data [8]. A point of contention is the possible data loss caused by the translation of sheep to human orthologs. The annotation of the sheep genome is improving but it cannot match the information available on human genes and gene functions, which is especially important when investigating biological processes [9, 63]. In this light, the correspondence between ‘human’ biological processes and ‘sheep’ histology further validates the study outcomes.

As mentioned, the effect of the FGF2-VEGF-functionalized collagen scaffolds on gene expression at two weeks post-implantation is subtle. It may be that the effects of COL-HEP/GF are more profound before this timepoint. Although somewhat protected by heparin, FGF2 and VEGF are expected to slowly release from the scaffold upon implantation. In addition, the wound environment has a high protein turnover rate, which leads to degradation of the scaffold and its components. The pro-regenerative growth factors likely initiate a response during the first stages of wound healing. However, considering that FGF2 and VEGF will influence the wound environment upon implantation, it may be that there are other biological processes preceding the ones identified here at two weeks.

Another point of interest is the temporal nature of the wound healing response. Generally, FGF2 and VEGF are predominantly active during the proliferation phase: in adults this phase starts after approximately two weeks, following the initial inflammatory response. The scaffolds are implanted at the start of wound healing, meaning they release FGF2 and VEGF during the inflammatory phase. The impact of these growth factors may be limited during inflammation. On the other hand, the effects may be more enhanced when their application can be matched to the respective healing phase. Regardless of the timing, the biological processes affected by the FGF2-VEGF-functionalized collagen scaffold at the two-week timepoint are a part of the pro-regenerative cascade that leads to enhanced skin regeneration postnatally. Future research into biomaterials for skin regeneration should consider the temporal nature of growth factor signaling: by further developing biomaterials that allow control over the space and time of growth factor administration their effects may be enhanced.

In conclusion, by taking a retrospective approach and looking at the biological processes that are involved early on during regenerative wound healing a novel perspective has formed. The pro-regenerative effects of collagen-heparin-FGF2-VEGF on wound healing were detectable at two weeks post implantation in a fetal sheep full thickness wound model. More importantly, the functionalized scaffold promotes a subtle shift in the wound environment where matrix deposition and cell signaling is tightly controlled. This study has provided a novel insight into (fetal) wound healing: influencing only a few key processes early on during fetal wound healing eventually culminates in a substantial increase in regenerative potential.

## Supplementary Information

Dataset S1 and S2 are available to download from Mendeley Data (10.17632/vsbfvc342x.1) under CC-BY 4.0 licensing. Below is the link to the electronic supplementary material.

Supplementary file1 (XLSX 272 KB)

Supplementary file1 (XLSX 187 KB)

## Data Availability

The original contributions presented in the study are included in the article; further inquiries can be directed to the corresponding authors.
